# Investigating the Use of Serious Games for Cancer Control Among Children and Adolescents: Scoping Review

**DOI:** 10.2196/58724

**Published:** 2024-07-10

**Authors:** Sunghak Kim, Paije Wilson, Olufunmilola Abraham

**Affiliations:** 1 National Cancer Survivorship Center National Cancer Control Institute National Cancer Center Goyang Republic of Korea; 2 Ebling Library School of Medicine and Public Health University of Wisconsin-Madison Madison, WI United States; 3 Social and Administrative Sciences Division School of Pharmacy University of Wisconsin-Madison Madison, WI United States

**Keywords:** serious games, cancer control, children, adolescents, scoping review, game, games, gaming, cancer, oncology, pediatric, pediatrics, paediatric, paediatrics, child, children, youth, adolescent, adolescents, teen, teens, teenager, teenagers, synthesis, review methods, review methodology, search, searches, searching, scoping

## Abstract

**Background:**

Effective health care services that meet the diverse needs of children and adolescents with cancer are required to alleviate their physical, psychological, and social challenges and improve their quality of life. Previous studies showed that serious games help promote people’s health. However, the potential for serious games to be used for successful cancer control for children and adolescents has received less attention.

**Objective:**

This scoping review aimed to map the use of serious games in cancer prevention and cancer care for children and adolescents, and provide future directions for serious games’ development and implementation within the context of cancer control for children and adolescents.

**Methods:**

This study followed a combination of the PRISMA-ScR (Preferred Reporting Items for Systematic Reviews and Meta-Analyses Extension for Scoping Reviews) and the JBI (Joanna Briggs Institute) framework for the conduct of scoping reviews. PubMed, CINAHL Plus Full Text, Scopus, Web of Science Core Collection, and American Psychological Association (APA) PsycINFO databases were used for the search.

**Results:**

From the initial 2750 search results, 63 papers were included in the review, with 28 quantitative, 14 qualitative, and 21 mixed method studies. Most of the studies were cancer care serious game papers (55/63, 87%) and a small number of studies were cancer prevention serious game papers (8/63, 13%). The majority of the included studies were published between 2019 and 2023 (cancer prevention: 5/8, 63%; cancer care: 35/55, 64%). The majority of the studies were conducted in Europe (cancer prevention: 3/8, 38%; cancer care: 24/55, 44%) and North America (cancer prevention: 4/8, 50%; cancer care: 17/55, 31%). Adolescents were the most represented age group in the studies’ participants (cancer prevention: 8/8, 100%; cancer care: 46/55, 84%). All (8/8, 100%) cancer prevention serious game papers included healthy people as participants, and 45 out of 55 (82%) cancer care serious game papers included patients with cancer. The majority of cancer prevention serious game papers addressed game preference as a target outcome (4/8, 50%). The majority of cancer care serious game papers addressed symptom management as a target outcome (28/55, 51%). Of the cancer care studies examining serious games for symptom management, the majority of the studies were conducted to treat psychological (13/55, 24%) and physical symptoms (10/55, 18%).

**Conclusions:**

This review shows both the growth of interest in the use of serious games for cancer control among children and adolescents and the potential for bias in the relevant literature. The diverse characteristics of the included papers suggest that serious games can be used in various ways for cancer control among children and adolescents while highlighting the need to develop and implement serious games in underrepresented areas.

## Introduction

### Cancer Control for Children and Adolescents

The efforts of cancer control to reduce the cancer burden, including interventions in cancer prevention and care, have reduced the prevalence of cancer and ameliorated its impacts on individuals and communities [1,2]. Additionally, with advances in medical technology, the survival rate of children and adolescents who contract cancer has been increasing [3]. However, cancer remains a life-threatening disease for children and adolescents and requires intensive treatment over a long period of time [4]. Practicing cancer prevention is the most efficient approach to avoid the significant physical and psychological burdens experienced during the diagnosis and treatment of cancer [5]. Adolescence, especially, is a critical stage to develop one’s cognitive ability and acquire new behavioral factors. Therefore, learning about cancer risks and cancer prevention methods during adolescence may significantly impact one’s future health status [6,7]. Nevertheless, cancer prevention knowledge and educational opportunities for children and adolescents are limited [6,8]. Moreover, young patients with cancer easily experience fatigue, pain, sleep disorders, and anxiety and have difficulties in establishing their values and interpersonal relationships while undergoing prolonged and intense treatment [9-11]. Systematic and continuous care that meets patients’ needs is crucial to helping them overcome such challenges and improve their quality of life [12-14]. Nonetheless, due to the smaller number of young patients with cancer compared to adult patients with cancer, their needs may not be prioritized in medical policy formulation or service provision [15-17]. More active industrial and research activities supporting the development and implementation of effective cancer control methods for children and adolescents should be undertaken.

### The Use of Serious Games

In the significantly growing field of digital health care, based on the rapid development of information and communication technology and computer technology, the potential for serious games to be used as a successful means of cancer control is being recognized [18,19]. Serious games are digital or computerized games used primarily for educational and training purposes rather than entertainment and amusement [20]. With the widespread availability of electronic devices such as computers, gaming consoles, and mobile devices, many users can easily access and enjoy serious games [20]. Additionally, they can experience more interactive, immersive, and engaging game-based learning through various serious game content [21]. By playing serious games, users may not only obtain enjoyable and immersive experiences but also enhance motivation, engagement, and learning outcomes. Users can also develop their skills in critical thinking, decision-making, problem-solving, social interaction, time management, and so on by actively exploring the serious game content in a safe environment without physical constraints [22-25]. Adaptive and personalized functions and immediate feedback offered by the game system also promote users’ continuous learning cycles while retaining user engagement [26,27]. Previous literature has shown the effectiveness of serious games designed with diverse objectives, such as improving retention of knowledge [28], pain relief [29], and medication adherence [20] in the context of various diseases.

While several positive outcomes have been associated with the use of serious games, there are a few side effects associated with the use of serious games as a health intervention tool. Previous studies have reported the possibility that the complex features of serious games may increase users’ mental workload, which may negatively impact learning [30]. Additionally, some studies have argued that the addictive nature of video games should not be overlooked in the use of serious games [31] (interestingly, though, at least one study has argued the opposite effect, stating that serious games can be used as a solution to game addiction issues [32]). These potential negative effects can be prevented through careful consideration of the user groups, purposes, and appropriate uses of serious games during their development process [33].

### Serious Games in the Context of Cancer Control in Children and Adolescents

Past research has also explored the relationship between serious games and young people in the context of cancer [34,35]. Adolescents, especially, tend to have excellent adaptability to new technologies and possess substantial knowledge and experience with games as compared to users of other age groups [36]. Given the research on adolescents’ engagement with video games [37] and studies conducted in game-based learning [38] and narrative persuasion [39-41], one may anticipate the positive influence of serious games on adolescents’ learning and persuasion outcomes. When it comes to cancer prevention, adolescents can learn about complex cancer concepts and relevant prevention and treatment methods by interacting with engaging characters and objects and actively performing game quests embedded in serious games [34]. Concerning cancer care, serious games can help distract teenage patients with cancer from the pain and anxiety associated with treatment, facilitating successful coping with the challenges of cancer [35]. Serious games can also provide psychological and social support or assist in promoting rehabilitation and physical activity [42]. However, more research is needed relating to the use of serious games in establishing successful strategies for pediatric cancer control. Cancer control for children and adolescents differs from that for adults, from the causes of cancer to the objectives and methods of cancer treatment [43,44]. For example, because children and adolescents are still cared for by caregivers (ie, legal guardians), not only young patients with cancer but also their caregivers should be included in the scope of cancer control [45]. This is just one of many characteristics indicating the need for different approaches and considerations when providing cancer control services for children and adolescents as compared to adults. Despite these unique needs, there is a relative lack of academic and industrial projects specifically addressing serious games within the context of cancer control in children and adolescents. Systematic and comprehensive consideration of existing studies can inform and direct future research in the use of serious games for cancer control in children and adolescents.

### Aim of This Study

This paper assesses the extent to which serious games have been used for cancer control (ie, cancer prevention and care) in children and adolescents. Specifically, this scoping review aimed to understand trends in serious games research in cancer prevention and cancer care for children and adolescents within published, original research papers, and investigate future directions for the application of serious games in successful cancer control for children and adolescents. Due to the importance of introducing cancer prevention and care in children and adolescents, and as the needs in the context of cancer prevention will differ from those of cancer care, this review also sought to compare serious games research focusing on cancer prevention with those focusing on cancer care to identify any key differences in research trends for these subjects.

This study will provide valuable insight and inform successful health intervention strategies relating to the use of serious games within the context of cancer control in children and adolescents. Considering the salience of cancer prevention education in adolescence, we also anticipate this study will encourage future research focusing on the use of serious games within this context.

## Methods

### Study Design

As the goal of the review was to explore and summarize the literature on our topic, which aligns with one of the primary purposes of a scoping review [46], a scoping review methodology was chosen for this study. Within the context of the population, concept, and context framework, our population was children and adolescents, our concept was serious games, and our context was cancer control. This study followed the JBI (Joanna Briggs Institute) framework for the conduct of scoping reviews, which specifically involved the following steps: “(1) defining and aligning the objectives and questions; (2) developing and aligning the inclusion criteria with the objectives and questions; (3) describing the planned approach to evidence searching selection, data extraction, and presentation of the evidence; (4) searching for the evidence; (5) selecting the evidence; (6) extracting the evidence; (7) analysis of the evidence; (8) presentation of the results; and (9) summarizing the evidence per the purpose of the review, making conclusions and noting any implications of the findings” [46]. This study was reported using the PRISMA-ScR (Preferred Reporting Items for Systematic Reviews and Meta-Analyses Extension for Scoping Reviews) [47]. The PRISMA-ScR checklist can be found in Multimedia Appendix 1. The protocol for this review was registered in the OSF (Open Science Framework) [48].

### Search Strategy

The literature search was developed by a health sciences librarian (PW) and included a combination of controlled vocabulary and keywords relating to serious games and cancer. No date, language, age, or geographical filters were applied to the search. The search was translated by PW for use in PubMed, CINAHL Plus Full Text (via EBSCOhost), Scopus, Web of Science Core Collection, and American Psychological Association (APA) PsycINFO (via EBSCOhost) databases. PW executed the search in each database on June 2, 2022, and reran the search using Bramer and Bain’s [49] method on December 15, 2023, to retrieve any new results since the date of the first search. The results of the search were imported into EndNote 20 (Clarivate) for the first search run and EndNote 21 (Clarivate) for the search rerun. The results were deduplicated using Bramer et al’s [50] method. The deduplicated results were then exported as a Microsoft Excel sheet (version 2402), which was used for screening. The full search strategy used for each database can be found in Multimedia Appendix 2.

### Study Selection

To be included in the scoping review, studies needed to meet the inclusion criteria outlined in Textbox 1.

In total, 2 authors (SK and PW) independently screened the titles and abstracts of the records based on the above eligibility criteria (ie, the authors reviewed each of the records in duplicate). Any conflicts were discussed and resolved via consensus. After the completion of the title and abstract screening, the process was repeated for the full-text screening, with the same 2 authors reviewing all records in duplicate based on the previously mentioned eligibility criteria, and all conflicts being discussed and resolved via consensus. In cases where conflicts were challenging to resolve, a third reviewer served as the tiebreaker (OA). All screening (including both title and abstract and full-text screening) was performed in a Microsoft Excel sheet (version 2402).

**Eligibility criteria.**

**Inclusion criteria**

- Language: the paper was written in English.
- Publication type: the paper was a full, original research paper (ie, primary study).
- Age:
  The study recruited participants who were aged 19 years or younger. The maximum age was based on the World Health Organization (WHO)’s definition of adolescents [51]. We also included studies where data were indirectly collected for this age group, such as when parents were interviewed about their children.
  If children or adolescents and adults were examined in the same study, the paper separately or predominantly reported on the findings of children or adolescent participants.
- Serious games used for cancer prevention or care:
  The study examined serious games being used for cancer prevention or care. Within the context of this study, we defined “serious games” as digital or computerized games that were used for education, behavior modification, or therapy. As a note, this definition was inclusive of digital or computerized entertainment games that were used for therapeutic purposes (eg, examining whether playing a commercial video game, such as Frogger, distracted patients from cancer-related symptoms [52]). For cancer prevention, this included studies that used serious games to educate or modify behaviors for cancer prevention. For cancer care, this included studies that used serious games to care for patients or survivors of cancer with cancer-related symptoms, helping them to overcome cancer-related challenges or educating them about their cancer diagnoses or treatments.
  For studies that examined using serious games in combination with other interventions for cancer prevention or care, the paper separately or predominantly reported on the impact of the serious games on cancer prevention or care.
  For studies that examined using serious games in the context of cancer and other diseases, the paper separately or predominantly reported on the impact of the serious games on cancer prevention or care.

**Exclusion criteria**

- Language: the paper was not written in English.
- Publication type: the paper was a literature review, editorial, commentary, essay, white paper, or a type of gray literature.
- Age:
  The study only recruited participants that were aged 20 years or older, and no data (direct or indirect) were collected on children or adolescents.
  If children or adolescents and adults were examined in the same study, the study predominantly consisted of adults and reported their findings cumulatively (ie, they did not separately report the findings of children or adolescent participants).
- Serious games used for cancer prevention or care:
  The study only examined nondigital games, such as board games.
  The study did not examine serious games being used for cancer prevention or care.
  For studies that examined using serious games in combination with other interventions for cancer prevention or care, the paper reported their findings cumulatively (ie, they did not separately report on their findings for the serious games).
  For studies that examined other diseases as well as cancer prevention or care, the study was not predominantly focused on cancer prevention or care and reported their findings cumulatively (ie, they did not separately report their findings for cancer prevention or care).

### Data Extraction

Following a full-text review, 2 authors (SK and PW) created and piloted a standardized extraction chart in Microsoft Excel (version 2402). The pilot entailed the 2 authors independently charting data from the first 10 included reports into the extraction chart, and meeting to identify areas that necessitated clarification or further standardization. After the pilot, information from all included studies was independently charted by the same authors into the extraction chart. The chart included each study’s authors, title, publication year, location, participant age (ie, whether participants were children, adolescents, or adults), participant type (ie, whether the participants were patients with cancer, survivors of cancer, health professionals, caregivers, or healthy people; or whether the participant type was unspecified), serious game objective (ie, whether the serious game was being used for cancer prevention or cancer care), serious game name, target outcome (ie, what outcomes were primarily examined for the study), and, for studies that had symptom management as a target outcome, target symptom (ie, what symptoms were primarily examined for the study). Any discrepancies in the data charting were discussed by the 2 authors and resolved via consensus.

## Results

### Overview

**Figure 1:**
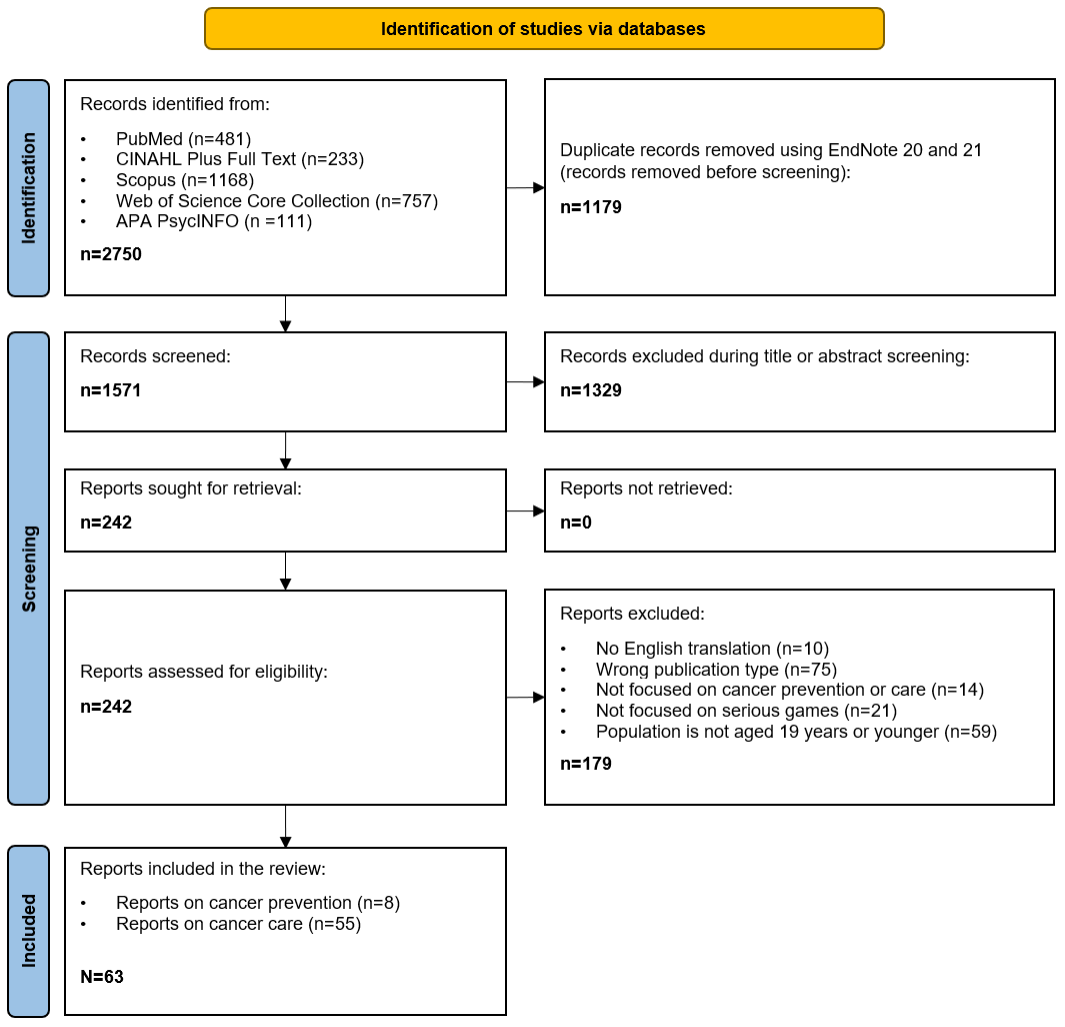
PRISMA flow diagram. Adapted from Page et al. APA: American Psychological Association; PRISMA: Preferred Reporting Items for Systematic Reviews and Meta-Analyses.

The search retrieved a total of 2750 records. Of those records, 1179 were identified as duplicates and removed. Title and abstract screening was performed on the remaining 1571 records, of which 1329 were excluded. The remaining 242 records underwent full-text screening, with 179 of these records being excluded (resulting in a total of 63 included records for the review; see Figure 1 [53]).

### Basic Information

Of the 63 papers included in this study, cancer care was the serious game objective with the most research (55/63, 87%) [35,42,52,54-105], with cancer prevention only having 8 papers (13%) [7,34,106-111]. The majority of the included studies were published between 2019 and 2023. To be specific, 5 out of 8 (63%) cancer prevention serious game papers [7,106,109-111] and 35 out of 55 (64%) cancer care serious game papers [35,42,54,58,59,61-63,65,66,71,72,74,75,78-82, 85,91-105] were published between 2019 and 2023. The studies were conducted in Asia, Europe, North America, and Oceania, with the majority of the studies being conducted in Europe and North America. Of the 8 cancer prevention serious game papers, 3 (38%) [107,109,110] and 4 (50%) [7,34,106,108] studies were conducted in Europe and North America, respectively. Of the 55 cancer care serious game papers, 24 (44%) [35,58, 61-63,65-67,70,74,75,81-83,85-87,92,93,97-100,103] and 17 (31%) [52,54-57,59,60,63,68,69,73,78,79,84,89,90,102] studies were conducted in Europe and North America, respectively. Of the 8 cancer prevention serious game studies, 3 (38%) were quantitative [34,110,111], 3 (38%) were qualitative [7,107,108], and 2 (25%) were mixed methods studies [106,109]. Of the 55 cancer care serious game studies, 25 (45%) were quantitative [35,42,52,54-56,58,64,69,71-74,76,77,80,84,87-89,91,96,97,100,105], 11 (20%) were qualitative [57,59,62,63,67,81,82,93,99,102,103], and 19 (35%) were mixed methods studies [60,61,65, 66,68,70,75,78,79,83,85,86,90,92,94,95,98,101,104]. For the distribution of these studies by publication year and location see Table 1.

**Table 1:** Basic information of included papers.

Characteristics		Serious game objective	
		Cancer prevention (n=8)	Cancer care (n=55)
**Publication year, n (%)^a^**			
	1985-1998 [52,73]	0 (0)	2 (4)
	1999-2003 [89]	0 (0)	1 (2)
	2004-2008 [55,56,68,69]	0 (0)	4 (7)
	2009-2013 [57,76,77,83,90,107]	1 (13)	5 (9)
	2014-2018 [34,60,64,67,70,84,86-88,108]	2 (25)	8 (15)
	2019-2023 [7,35,42,54,58,59,61-63,65,66,71,72,74,75,78-82,85,91-106,109-111]	5 (63)	35 (64)
**Location, n (%)^a^**			
	Asia [42,64,71,72,76,77,80,88,91,94-96,101,105,111]	1 (13)	14 (26)
	Europe [35,58,61-63,65-67,70,74,75,81-83,85-87,92,93,97-100,103,107,109,110]	3 (38)	24 (44)
	North America [7,34,52,54-57,59,60,63,68,69,73,78,79,84,89,90,102,106,108]	4 (50)	17 (31)
	Oceania [55,56,69,104]	0 (0)	4 (7)
**Study type, n (%)^a^**			
	Quantitative [34,35,42,52,54-56,58,64,69,71-74,76,77,80,84,87-89,91,96,97,100,105,110,111]	3 (38)	25 (45)
	Qualitative [7,57,59,62,63,67,81,82,93,99,102,103,107,108]	3 (38)	11 (20)
	Mixed methods [60,61,65,66,68,70,75,78,79,83,85,86,90,92,94,95,98,101,104,106,109]	2 (25)	19 (35)

^a^Note that the frequency numbers may not add up to the total number of studies (N=63), as some studies included more than one category. Percentages may also not add up to 100% due to rounding.

### Participant Characteristics

Children and adolescents were the main participants represented in the papers included in this review, which aligned with this study’s purpose. The adolescents’ age group had the greatest representation in the included studies—all (8/8, 100%) cancer prevention serious game papers [7,34,106-111] and 46 out of 55 (84%) cancer care serious game papers [35,42,52,54-62, 64-66,68-80,83,84,86-93,95-98,100,101,104,105] involved adolescents as participants. Interestingly, many studies included adult participants as well as adolescents or children. As mentioned in our inclusion criteria, studies examining adult populations were included if these also examined and isolated data for our population of interest (ie, children or adolescents) or if adults were interviewed about children or adolescents. Of our included studies, 25 of the 55 (45%) cancer care papers [52,55,56,60-62,66-69,79-82,84,85,92-95,97,99,101,102,104] and 2 of the 8 (25%) cancer prevention papers [34,109] included adults as well as adolescents or children. Further, 2 studies only included adults (being studies where parents were interviewed about their children, but where the children did not participate in the study themselves), with both of these studies being cancer care papers [63,103].

All (8/8, 100%) cancer prevention serious game papers included healthy people as participants [7,34,106-111]. Regarding cancer care serious game papers, 45 out of 55 (82%) studies included patients with cancer [35,42,52,55-57,59-62,64-66,68-79,81-84, 88-90,92-102,104,105]. Surprisingly, several papers also involved health professionals and caregivers as participants, wherein these groups were interviewed to solicit their impressions and opinions of how the serious games affected their young patients or children, respectively [60-63,66,79, 81,82,85,92-95,97,99,101-103]. In these cases, where health professionals’ or caregivers’ opinions or quotes were associated with our population of interest (ie, children or adolescents) and these opinions or quotes were included in the results section of the studies, our review study included those papers and counted the health professionals and caregivers as the study’s participants.

Table 2 presents more details about the distribution of included studies by participant age and participant type.

**Table 2:** Participant characteristics of included papers.

Characteristics		Serious game objective	
		Cancer prevention (n=8)	Cancer care (n=55)
**Participant age (years), n (%)^a^**			
	Children (0-9) [35,42,52,57-62,64-67,70,72,74,76-79,81-87,91-99,101,102,107,108]	2 (25)	38 (69)
	Adolescents (10-19) [7,34,35,42,52,54-62,64-66,68-80,83,84,86-93,95-98,100,101,104-111]	8 (100)	46 (84)
	Adults (≥20) [34,52,55,56,60-63,66-69,79-82,84,85,92-95,97,99,101-104,109]	2 (25)	27 (49)
**Participant type, n (%)^a^**			
	Patients with cancer [35,42,52,55-57,59-62,64-66,68-79,81-84,88-90,92-102,104,105]	0 (0)	45 (82)
	Survivors of cancer [54,57,58,80,86,87,91]	0 (0)	7 (13)
	Health professionals [60,63,66,81,82,85,93,94,97]	0 (0)	9 (16)
	Caregivers [60-63,66,79,81,82,92,95,97,99,101-103]	0 (0)	15 (27)
	Healthy people [7,34,66,70,75,85,91,106-111]	8 (100)	5 (9)
	Unspecified (not disclosed) [67]	0 (0)	1 (2)

^a^Note that the frequency numbers may not add up to the total number of studies (N=63), as some studies included more than one category. Percentages may also not add up to 100% due to rounding.

### Role of Serious Games in Cancer Control for Young People

The papers included in this review examined the use of serious games for a variety of target outcomes. For cancer prevention, the target outcome most addressed by the papers was exploring participants’ preferences for the content of the serious games (ie, participants’ satisfaction with the serious game or likes and dislikes about features within the serious game; 4/8, 50%) [7,108,110,111], followed by educating participants about cancer (3/8, 38%) [34,106,109], and promoting healthy behaviors (1/8, 13%) [107]. For cancer care, the target outcome most addressed by the papers was using serious games for symptom management (28/55, 51%) [35,42,52,58,61,64,65,71-73,76,77, 81-84,86-89,91,92,94,97,99,100,104,105], followed closely by exploring participants’ preferences for the content of the serious games (24/55, 44%) [55,56,62,63,67,68,70,75,79,84-86, 90,92-98,101-104]. Other target outcomes observed for cancer care included promoting healthy behaviors (10/55, 18%) [42,54,57,60,65,74,80,95,96,101], symptom reporting (7/55, 13%) [59,66,75,78,79,90,102], cancer education (6/55, 11%) [55,56,80,95,101,105], and treatment adherence (3/55, 6%) [69,95,101]. Of the cancer care studies examining serious games for symptom management, the majority of the studies were conducted to treat psychological (13/55, 24%) [61,71-73,76,77, 81,83,89,92,94,97,100] and physical symptoms (10/55, 18%) [35,42,52,58,65,81,83,86,99,104]. Naturally, none of the cancer prevention serious game papers were conducted with the goal of managing cancer-related symptoms.

Table 3 presents more details about the distribution of included studies by target outcome and target symptom.

**Table 3:** The role of serious games in included papers.

Characteristics		Serious game objective	
		Cancer prevention (n=8)	Cancer care (n=55)
**Target outcome, n (%)^a^**			
	Serious game preference [7,55,56,62,63,67,68,70,75,79,84-86,90,92-98,101-104,108,110,111]	4 (50)	24 (44)
	Treatment adherence [69,95,101]	0 (0)	3 (6)
	Cancer education [34,55,56,80,95,101,105,106,109]	3 (38)	6 (11)
	Healthy behavior promotion [42,54,57,60,65,74,80,95,96,101,107]	1 (13)	10 (18)
	Symptom management [35,42,52,58,61,64,65,71-73,76,77,81-84,86-89,91,92,94,97,99,100,104,105]	0 (0)	28 (51)
	Symptom reporting [59,66,75,78,79,90,102]	0 (0)	7 (13)
**Target symptom, n (%)^a^**			
	Psychological symptoms^b^ [61,71-73,76,77,81,83,89,92,94,97,100]	0 (0)	13 (24)
	Physical symptoms^c^ [35,42,52,58,65,81,83,86,99,104]	0 (0)	10 (18)
	Cognitive symptoms^d^ [42,58,84,87,88,104]	0 (0)	6 (11)
	General side effects^e^ [73,105]	0 (0)	2 (4)
	Other^f^ [64,82,87,91,104,105]	0 (0)	6 (11)

^a^Note that the frequency numbers may not add up to the total number of studies (N=63), as some studies included more than one category. Percentages may also not add up to 100% due to rounding.

^b^“Psychological symptoms” includes papers that addressed “anxiety,” “depression,” “distress,” “emotional state,” “psychosocial symptoms,” or “psychological symptoms” in this study.

^c^“Physical symptoms” includes papers that addressed “endurance,” “fatigue,” “motor performance,” “nausea,” “pain,” or “physical activity” in this study.

^d^“Cognitive symptoms” includes papers that addressed “cognitive behavioral effects,” “cognitive function,” “functional capacity,” or “reading deficits” in this study.

^e^“General side effects” includes papers that addressed “side effects of chemotherapy” or “symptoms” (nonspecific) in this study.

^f^“Other” includes papers that addressed “activities of daily living,” “daily performance,” “quality of life,” or “sleep” in this study.

## Discussion

### Overview

This scoping review aimed to examine the extent to which serious games have been used within the context of cancer prevention and care for children and adolescents within published, original research papers, and investigate future directions for the application of serious games in successful cancer control for children and adolescents. In this review, we identified papers that explored the potential of serious games being used for successful cancer control for children and adolescents. When observing the distribution of these papers in a comprehensive map, we also identified several gaps that may introduce bias into the existing literature.

### Need for Cancer Prevention Serious Games Research

The results of this study showed that there were considerably fewer cancer prevention serious game papers than cancer care serious game papers. This difference may indicate that cancer prevention serious games are perceived as being less important than cancer care serious games. The necessity of pediatric cancer prevention might also be overlooked because of the relatively insufficient medical infrastructure and interest in pediatric cancer prevention as compared to adult cancer prevention, being in part due to the smaller number of pediatric patients with cancer compared to adult patients with cancer [15-17]. Another explanation could be the complex etiology of pediatric cancer. For adult cancers, there are several studies establishing the associations between cancer occurrence and lifestyle or environmental risk factors [112,113], which may help people take preventive measures. However, it is known that lifestyle or environmental risk factors are unlikely to play a significant role in the occurrence of pediatric cancers [114]. The lack of evidence about lifestyle or environmental risk factors may hinder identifying them and reduce preventive efforts for pediatric cancers. Consequently, the awareness of pediatric cancer prevention may be low. Nevertheless, considering that cancer prevention interventions are the most effective way to reduce cancer-related risks, it is essential to develop and provide cancer prevention services for pediatric cancers. Future research should be conducted to develop serious games that educate young people about cancer so that they can have knowledge about cancer and preemptively engage in healthy behaviors.

### Disparities in Cancer Control Serious Games Research Relating to the Publication Year and Location

This study’s findings also confirmed that the publication year and location are concentrated in specific years and countries. Both cancer prevention serious game papers and cancer care serious game papers were largely published after the year 2019. This distribution may imply a trend that researchers are recently paying more attention to the application of serious games in digital health care [115]. The impact of serious games will be amplified by systematically delving into the conceptual and theoretical underpinnings of serious games and uncovering strategies to develop and use serious games for constructive and socially beneficial purposes. Future research needs to investigate the positive influence of serious games and accelerate their implementation in cancer prevention and care. Doing so will facilitate the successful use of serious games in pediatric cancer control and spearhead advancements in digital health care. Additionally, research on serious games in both cancer prevention and cancer care has predominantly been conducted in Europe and North America. As the positive effects of serious games in the medical field have been indicated through existing research, future research needs to explore avenues to encourage serious game studies in the medical field for researchers in other geographic regions. Such efforts would be beneficial to mitigate disparities in digital technology and reduce inequalities in access to health care services.

### Embracing Various Age Groups in Cancer Control Serious Games Research

Moreover, our study revealed distinct patterns in the distribution of papers based on participant characteristics. Regarding participant age, it was observed that all papers on cancer prevention serious games focused on adolescents. Similarly, the predominant age group featured in papers concerning cancer care serious games was also adolescents. These findings are not unexpected, considering adolescents’ familiarity with gaming [36] and the research objective of this review study. However, the observed distribution indirectly suggests a divergence between the child and adolescent groups, while indicating a relative neglect in research focusing on serious games for children. In addition to exploring cancer control serious games tailored for children, future studies could also compare the characteristics and uses of serious games designed for children with those designed for adolescents, and, in doing so, identify salient differences between the 2 groups. An additional finding of note was that, due to the intricate dynamics of pediatric cancer that affect not only young patients but also adults closely associated with them [45], certain studies incorporated adult participants as well as children or adolescents. More research should encompass both child and adult users when developing a serious game for effective pediatric cancer control or when evaluating user experiences, as doing so could furnish more comprehensive and nuanced findings.

### Embracing Various Participant Types in Cancer Care Serious Games Research

Regarding participant type, it was observed that all papers on cancer prevention serious games exclusively targeted healthy people. Conversely, the primary participant type featured in papers focusing on cancer care serious games was patients with cancer. Notably, cancer care serious game papers included a diverse array of participant types, with some studies even including multiple participant types. This suggests the potential for serious games to cater to diverse participant types, even those characterized by different interests and attributes. Future research should strive to understand the different needs of multiple participant types to develop effective, and far-reaching cancer care serious games. A comprehensive approach to identify different user needs within the context of serious games should also be encouraged.

The diverse array of participant types also implies the potential for participants to play a variety of roles in developing serious games. Pediatric patients with cancer and survivors can share their opinions and user experiences when using serious games [55,86]. Caregivers can also share their opinions and user experiences when they or their dependents use serious games while also encouraging their dependents to play serious games [60,61]. Health professionals can provide expertise and a nuanced understanding of the needs of their patients and can therefore provide invaluable feedback for the design and content of serious games [60,82]. All these roles can facilitate the evaluation and development of serious games.

Finally, our review found that a small number of papers included survivors of cancer, caregivers, or health professionals as participants when examining the use of serious games for cancer control in children or adolescents. In particular, survivors of cancer and caregivers have difficulties in resolving their unmet needs or draw less emphasis or attention to cancer care services [116,117]. Crafting appropriate serious games for survivors of cancer and caregivers based on an advanced understanding of them may improve both the quantity and quality of cancer care services for young people. As health care providers, health professionals can be overlooked in their potential to play a role in serious game research for cancer control in children or adolescents; however, as stated previously, health professionals can provide invaluable feedback for the development and evaluation of serious games.

### Enriching Cancer Control Serious Games Research by Focusing on Underrepresented Target Outcomes

The roles of the serious games differed between cancer prevention and cancer care. Serious game preference was one of the main target outcomes for both cancer prevention serious game papers and cancer care serious game papers. Cancer education emerged as another key target outcome for cancer prevention serious game papers, whereas symptom management took precedence in cancer care serious game papers. Similar to the case with participant types, cancer care serious game papers addressed a diverse range of target outcomes, with some studies addressing multiple target outcomes within a single investigation. This suggests the feasibility of developing and deploying serious games for young people’s cancer care with multifaceted purposes. There were, however, some target outcomes that were underrepresented, such as healthy behavior promotion for cancer prevention studies and management of cognitive symptoms for cancer care studies. Future research should delve into effective strategies for serious game development and implementation in these underrepresented target outcomes, thus bridging existing gaps and fostering more comprehensive discussions concerning the use of serious games in cancer prevention and care.

### Limitations

This study demonstrated the research state on the use of serious games for cancer control among children and adolescents and suggested the future directions of serious game development and research; however, this study did have limitations. First, only papers written in English were included in the review, so valuable data from relevant papers published in other languages may have been excluded. Second, multiple studies included in this study’s data analysis recruited not only children and adolescents but also adults as participants; therefore, careful interpretation of serious games’ influence on children and adolescents is necessary. Third, we opted to limit our results to a reputable and manageable selection, with the goal of the review being to provide an overview of original, primary studies on our topic. Due to this, we refrained from including paper types such as reviews, conference proceedings, etc, in our review. Fourth, we did not evaluate the statistical outcomes of the serious games within each study, as this was not within the scope of our review (with the scope of our review being to map the use of serious games within the context of cancer control). Future research, such as a systematic review, could provide valuable insight into the statistical efficacy of serious games within focused areas of cancer control. Future studies could also assess topics such as features and frameworks of serious games, providing more insight into their development.

### Conclusions

This review study shows that there has been an increased interest in the use of serious games for cancer control among children and adolescents. At the same time, this study reveals that the number of papers has been skewed in terms of the purpose and context of serious games for cancer control in children and adolescents, with cancer prevention serious games in young people having received considerably less attention than those of cancer care. Additionally, the frequencies of study participants’ characteristics and target outcomes differ depending on the serious game objectives. Regarding cancer prevention serious game papers, these studies primarily examined serious game preference. Regarding cancer care serious game papers, these studies primarily examined serious game preference and how serious games help alleviate cancer-related symptoms. These differences suggest that serious games can be used in multiple ways within cancer control in children and adolescents while highlighting the need to develop and implement serious games in underrepresented areas. Further studies are needed to comprehensively examine which features of serious games enhance young people’s cancer control and what evidence-based and theory-driven methods are available to develop effective serious games for this purpose. Integrating these future findings into this review study’s outcomes may help advance successful serious game development and implementation for young people’s cancer control.

## Appendix

Multimedia Appendix 1 The PRISMA-ScR (Preferred Reporting Items for Systematic Reviews and Meta-Analyses Extension for Scoping Reviews) checklist of this scoping review.

Multimedia Appendix 2 The searching strategy of this scoping review.

## Abbreviations

APA: American Psychological Association
JBI: Joanna Briggs Institute
OSF: Open Science Framework
PRISMA-ScR: Preferred Reporting Items for Systematic Reviews and Meta-Analyses Extension for Scoping Reviews

## Data Availability

The data sets generated or analyzed during this study are available from the corresponding author upon reasonable request.
