# Supplementary material for: Investigating the Use of Serious Games for Cancer Control Among Children and Adolescents: Scoping Review
Source: JMIR Serious Games. 2024 Jul 10;12:e58724. doi: 10.2196/58724 (PMC11269965; doi:10.2196/58724)
Supplement: Multimedia Appendix 2 [file games_v12i1e58724_app2.pdf]

Search Strategies

Results Overview:

Searches ran on 06/02/2022 and again on 12/15/2023. The results from 12/15/2023 are displayed below.

Database	Results
PubMed	481
CINAHL Plus Full Text (EBSCOhost)	233
Scopus	1,168
Web of Science Core Collection*	757
PsycINFO (EBSCOhost)	111

Total results before deduplication: 2,750

Total results after deduplication: 1,571

1,179 duplicates were found using Bramer et al.'s (2016) "De-duplication of database search results for systematic reviews in EndNote" method (citation below).

Bramer WM, Giustini D, de Jonge GB, Holland L, Bekhuis T. De-duplication of database search results for systematic reviews in EndNote [published correction appears in J Med Libr Assoc. 2017 Jan;105(1):111]. *J Med Libr Assoc.* 2016;104(3):240-243. doi:10.3163/1536-5050.104.3.014

*** The Web of Science Core Collection included the following:**

- Science Citation Index Expanded (SCI-EXPANDED)
- Social Sciences Citation Index (SSCI)
- Arts & Humanities Citation Index (AHCI)
- Conference Proceedings Citation Index – Science (CPCI-S)
- Conference Proceedings Citation Index – Social Science & Humanities (CPCI-SSH)
- Book Citation Index – Science (BKCI-S)
- Book Citation Index – Social Sciences & Humanities (BKCI-SSH)
- Emerging Sources Citation Index (ESCI)
- Current Chemical Reactions (CCR-EXPANDED)
- Index Chemicus (IC)

Contents

Results Overview: ... 1

PubMed: 395 results ... 3

PubMed Block Format: ... 3

PubMed Line by Line Format: ... 5

CINAHL Plus Full Text (EBSCOhost): 223 results ... 6

CINAHL Plus Full Text (EBSCOhost) Block Format: ... 6

CINAHL Plus Full Text (EBSCOhost) Line by Line Format: ... 8

Scopus: 985 results ... 9

Scopus Block Format ... 9

Scopus Line by Line Format ... 10

Web of Science Core Collection: 638 results ... 11

Web of Science Core Collection Block Format: ... 11

Web of Science Core Collection Line by Line Format: ... 12

PsycINFO (EBSCOhost): 103 results ... 13

PsycINFO (EBSCOhost) Block Format: ... 13

PsycINFO (EBSCOhost) Line by Line Format: ... 15

PubMed: 481 results (12/15/2023)

PubMed Original Search in Block Format:

("Video games"[mesh] OR "serious game"[tiab] OR "serious games"[tiab] OR "serious gaming"[tiab] OR "applied game"[tiab] OR "applied games"[tiab] OR "applied gaming"[tiab] OR exergam*[tiab] OR "exer gaming"[tiab] OR "exer game*"[tiab] OR Nintendo*[tiab] OR Wii[tiab] OR Wiis[tiab] OR playstation*[tiab] OR gamecube*[tiab] OR oculus[tiab] OR Kinect[tiab] OR Kinects[tiab] OR Gameboy*[tiab] OR "game boy*"[tiab] OR videogam*[tiab] OR "technology game*"[tiab] OR "game program*"[tiab] OR "gaming program*"[tiab] OR "gaming technolog*"[tiab] OR "game technolog*"[tiab] OR "electronic game*"[tiab] OR "electronic gaming"[tiab] OR "gaming electronic*"[tiab] OR "game electronic*"[tiab] OR (("virtual reality"[mesh] OR "augmented reality"[mesh] OR video[tiab] OR "mixed realit*"[tiab] OR "AR"[tiab] OR "VR"[tiab] OR mobile[tiab] OR iphone*[tiab] OR smartphone*[tiab] OR phone[tiab] OR phones[tiab] OR cellphone*[tiab] OR digital[tiab] OR virtual[tiab] OR augmented[tiab] OR computer*[tiab] OR console[tiab] OR consoles[tiab] OR interactive[tiab] OR handheld*[tiab] OR "hand held*"[tiab] OR "PC"[tiab] OR "PCs"[tiab] OR desktop[tiab] OR ipad[tiab] OR ipads[tiab] OR online[tiab] OR "on line"[tiab] OR internet[tiab] OR immers*[tiab] OR device*[tiab] OR xbox[tiab] OR Xboxes[tiab] OR audiovisual[tiab] OR "audio visual"[tiab] OR laptop*[tiab] OR tablet[tiab] OR tablets[tiab]) AND (game[tiab] OR games[tiab] OR gaming*[tiab] OR gamification*[tiab] OR gamify*[tiab] OR gamifie*[tiab] OR gamer*[tiab]))) AND (Neoplasms[mesh] OR "cancer survivors"[mesh] OR "medical oncology"[mesh] OR "oncology service, hospital"[mesh] OR "oncology nursing"[mesh] OR "cancer pain"[mesh] OR "integrative oncology"[mesh] OR cancer*[tiab] OR carcinoma*[tiab] OR oncolog*[tiab] OR psychooncolog*[tiab] OR neoplas*[tiab] OR tumor*[tiab] OR tumour*[tiab] OR malign*[tiab])

PubMed Revised Search in Block Format:

Note: This search was adapted from its original version (see the previous section, “PubMed Original Search", for the original version). The search was adapted to include proximity searching, being a feature introduced to PubMed in 2022 (citation below). 6 additional keywords and 2 MeSH terms were also added. All changes are indicated with yellow highlighting.

Citation: National Library of Medicine. PubMed Update: Proximity Search Now Available in PubMed. NLM Technical Bulletin. November 29, 2022. Accessed August 10, 2023. https://www.nlm.nih.gov/pubs/techbull/nd22/nd22_pubmed_proximity_search_available.html

("Video games"[mesh] OR "serious game"[tiab] OR "serious games"[tiab] OR "serious gaming"[tiab] OR "applied game"[tiab] OR "applied games"[tiab] OR "applied gaming"[tiab] OR exergam*[tiab] OR "exer gaming"[tiab] OR "exer game*"[tiab] OR Nintendo*[tiab] OR Wii[tiab] OR Wiis[tiab] OR playstation*[tiab] OR gamecube*[tiab] OR oculus[tiab] OR Kinect[tiab] OR Kinects[tiab] OR Gameboy*[tiab] OR "game boy*"[tiab] OR videogam*[tiab] OR computergame*[tiab] OR “technology game”[tiab:~2] OR “technology games”[tiab:~2] OR “technology gaming”[tiab:~2] OR “technologies game”[tiab:~2] OR “technologies games”[tiab:~2] OR “technologies gaming”[tiab:~2] OR “program game”[tiab:~2] OR “program games”[tiab:~2] OR “program gaming”[tiab:~2] OR “programs game”[tiab:~2] OR “programs games”[tiab:~2] OR “programs gaming”[tiab:~2] OR “electronic game”[tiab:~2] OR “electronic games”[tiab:~2] OR “electronic gaming”[tiab:~2] OR “electronics game”[tiab:~2] OR “electronics games”[tiab:~2] OR “electronics gaming”[tiab:~2] OR (("virtual reality"[mesh] OR "augmented reality"[mesh] OR video[tiab] OR "mixed realit*"[tiab] OR "AR"[tiab] OR "VR"[tiab] OR mobile[tiab] OR iphone*[tiab] OR smartphone*[tiab] OR phone[tiab] OR phones[tiab] OR cellphone*[tiab] OR digital[tiab] OR virtual[tiab] OR augmented[tiab] OR computer*[tiab] OR console[tiab] OR consoles[tiab] OR interactive[tiab] OR handheld*[tiab] OR "hand held*"[tiab] OR "PC"[tiab] OR "PCs"[tiab] OR desktop[tiab] OR ipad[tiab] OR ipads[tiab] OR online[tiab] OR "on line"[tiab] OR internet[tiab] OR immers*[tiab] OR device*[tiab] OR xbox[tiab] OR Xboxes[tiab] OR audiovisual[tiab] OR "audio visual"[tiab] OR laptop*[tiab] OR tablet[tiab] OR tablets[tiab] OR “smart glass*”[tiab] OR smartglass*[tiab] OR “google glass*”[tiab] OR mhealth[tiab] OR “m health”[tiab]) AND (game[tiab] OR games[tiab] OR gaming*[tiab] OR gamification*[tiab] OR gamify*[tiab] OR gamifie*[tiab] OR gamer*[tiab]))) AND (Neoplasms[mesh] OR "cancer survivors"[mesh] OR "medical oncology"[mesh] OR "oncology service, hospital"[mesh] OR "oncology nursing"[mesh] OR "cancer pain"[mesh] OR "integrative oncology"[mesh] OR “early detection of cancer”[mesh] OR “cancer vaccines”[mesh] OR cancer*[tiab] OR carcinoma*[tiab] OR oncolog*[tiab] OR psychooncolog*[tiab] OR neoplas*[tiab] OR tumor*[tiab] OR tumour*[tiab] OR malign*[tiab])

PubMed Revised Search in Line by Line Format:

Note: Blue cells indicate results for a complete concept

Search No.	Search String	Results
#1	"Video games"[mesh]	7,456
#2	"serious game"[tiab] OR "serious games"[tiab] OR "serious gaming"[tiab] OR "applied game"[tiab] OR "applied games"[tiab] OR "applied gaming"[tiab] OR exergam*[tiab] OR "exer gaming"[tiab] OR "exer game*"[tiab] OR Nintendo*[tiab] OR Wii[tiab] OR Wiis[tiab] OR playstation*[tiab] OR gamecube*[tiab] OR oculus[tiab] OR Kinect[tiab] OR Kinects[tiab] OR Gameboy*[tiab] OR "game boy*"[tiab] OR videogam*[tiab] OR computergame*[tiab]	7,764
#3	“technology game”[tiab:~2] OR “technology games”[tiab:~2] OR “technology gaming”[tiab:~2] OR “technologies game”[tiab:~2] OR “technologies games”[tiab:~2] OR “technologies gaming”[tiab:~2] OR “program game”[tiab:~2] OR “program games”[tiab:~2] OR “program gaming”[tiab:~2] OR “programs game”[tiab:~2] OR “programs games”[tiab:~2] OR “programs gaming”[tiab:~2] OR “electronic game”[tiab:~2] OR “electronic games”[tiab:~2] OR “electronic gaming”[tiab:~2] OR “electronics game”[tiab:~2] OR “electronics games”[tiab:~2] OR “electronics gaming”[tiab:~2]	1,099
#4	#1 OR #2 OR #3	13,790
#5	"virtual reality"[mesh] OR "augmented reality"[mesh]	7,201
#6	video[tiab] OR "mixed realit*"[tiab] OR "AR"[tiab] OR "VR"[tiab] OR mobile[tiab] OR iphone*[tiab] OR smartphone*[tiab] OR phone[tiab] OR phones[tiab] OR cellphone*[tiab] OR digital[tiab] OR virtual[tiab] OR augmented[tiab] OR computer*[tiab] OR console[tiab] OR consoles[tiab] OR interactive[tiab] OR handheld*[tiab] OR "hand held*"[tiab] OR "PC"[tiab] OR "PCs"[tiab] OR desktop[tiab] OR ipad[tiab] OR ipads[tiab] OR online[tiab] OR "on line"[tiab] OR internet[tiab] OR immers*[tiab] OR device*[tiab] OR xbox[tiab] OR Xboxes[tiab] OR audiovisual[tiab] OR "audio visual"[tiab] OR laptop*[tiab] OR tablet[tiab] OR tablets[tiab] OR “smart glass*”[tiab] OR smartglass*[tiab] OR “google glass*”[tiab] OR mhealth[tiab] OR “m health”[tiab]	2,072,695
#7	#5 OR #6	2,072,967
#8	game[tiab] OR games[tiab] OR gaming*[tiab] OR gamification*[tiab] OR gamify*[tiab] OR gamifie*[tiab] OR gamer*[tiab]	53,879
#9	#7 AND #8	19,633
**#10**	**#4 OR #9**	**26,096**
#11	Neoplasms[mesh] OR "cancer survivors"[mesh] OR "medical oncology"[mesh] OR "oncology service, hospital"[mesh] OR "oncology nursing"[mesh] OR "cancer pain"[mesh] OR "integrative oncology"[mesh] OR “early detection of cancer”[mesh] OR “cancer vaccines”[mesh]	3,922,791
#12	cancer*[tiab] OR carcinoma*[tiab] OR oncolog*[tiab] OR psychooncolog*[tiab] OR neoplas*[tiab] OR tumor*[tiab] OR tumour*[tiab] OR malign*[tiab]	4,149,287
**#13**	**#11 OR #12**	**5,202,205**
**#13**	**#10 AND #13**	**481**

CINAHL Plus Full Text (EBSCOhost): 233 results (12/15/2023)

CINAHL Plus Full Text (EBSCOhost) Original Search in Block Format:

(MH "video games+" OR TI "serious game" OR AB "serious game" OR TI "serious games" OR AB "serious games" OR TI "serious gaming" OR AB "serious gaming" OR TI "applied game" OR AB "applied game" OR TI "applied games" OR AB "applied games" OR TI "applied gaming" OR AB "applied gaming" OR TI exergam* OR AB exergam* OR TI "exer gaming" OR AB "exer gaming" OR TI "exer game*" OR AB "exer game*" OR TI Nintendo* OR AB Nintendo* OR TI Wii OR AB Wii OR TI Wiis OR AB Wiis OR TI playstation* OR AB playstation* OR TI gamecube* OR AB gamecube* OR TI oculus OR AB oculus OR TI Kinect OR AB Kinect OR TI Kinects OR AB Kinects OR TI Gameboy* OR AB Gameboy* OR TI "game boy*" OR AB "game boy*" OR TI videogam* OR AB videogam* OR TI (technolog* N2 (game OR games OR gaming)) OR AB (technolog* N2 (game OR games OR gaming)) OR TI (program* N2 (game OR games OR gaming)) OR AB (program* N2 (game OR games OR gaming)) OR TI (electronic* N2 (game OR games OR gaming)) OR AB (electronic* N2 (game OR games OR gaming)) OR ((TI game OR AB game OR TI games OR AB games OR TI gaming* OR AB gaming* OR TI gamification* OR AB gamification* OR TI gamify* OR AB gamify* OR TI gamifie* OR AB gamifie* OR TI gamer* OR AB gamer*) AND (MH "virtual reality" OR MH "augmented reality" OR TI video OR AB video OR TI "mixed realit*" OR AB "mixed realit*" OR TI "AR" OR AB "AR" OR TI "VR" OR AB "VR" OR TI mobile OR AB mobile OR TI iphone* OR AB iphone* OR TI smartphone* OR AB smartphone* OR TI phone OR AB phone OR TI phones OR AB phones OR TI cellphone* OR AB cellphone* OR TI digital OR AB digital OR TI virtual OR AB virtual OR TI augmented OR AB augmented OR TI computer* OR AB computer* OR TI console AB console OR TI consoles OR AB consoles OR TI interactive AB interactive OR TI handheld* OR AB handheld* OR TI "hand held*" OR AB "hand held*" OR TI "PC" OR AB "PC" OR TI "PCs" OR AB "PCs" OR TI desktop OR AB desktop OR TI ipad OR AB ipad OR TI ipads OR AB ipads OR TI online OR AB online OR TI "on line" OR AB "on line" OR TI internet OR AB internet OR TI immers* OR AB immers* OR TI device* OR AB device* OR TI xbox OR AB xbox OR TI Xboxes OR AB Xboxes OR TI audiovisual OR AB audiovisual OR TI "audio visual" OR AB "audio visual" OR TI laptop* OR AB laptop* OR TI tablet OR AB tablet OR TI tablets OR AB tablets))) AND (MH (neoplasms+ OR "cancer fatigue" OR "cancer survivors" OR "cancer patients" OR "cancer pain" OR oncology+ OR "oncologic nursing+" OR "oncology care units" OR "rehabilitation, cancer" OR "oncologic care+") OR TI (cancer* OR carcinoma* OR oncolog* OR psychooncolog* OR neoplas* OR tumor* OR tumour* OR malign*) OR AB (cancer* OR carcinoma* OR oncolog* OR psychooncolog* OR neoplas* OR tumor* OR tumour* OR malign*))

CINAHL Plus Full Text (EBSCOhost) Revised Search in Block Format

Note: This search was adapted from its original version (see the previous section, “CINAHL Plus Full Text Original Search", for the original version). 6 additional keywords and 2 subject headings were added. All changes are indicated with yellow highlighting.

(MH "video games+" OR TI "serious game" OR AB "serious game" OR TI "serious games" OR AB "serious games" OR TI "serious gaming" OR AB "serious gaming" OR TI "applied game" OR AB "applied game" OR TI "applied games" OR AB "applied games" OR TI "applied gaming" OR AB "applied gaming" OR TI exergam* OR AB exergam* OR TI "exer gaming" OR AB "exer gaming" OR TI "exer game*" OR AB "exer game*" OR TI Nintendo* OR AB Nintendo* OR TI Wii OR AB Wii OR TI Wiis OR AB Wiis OR TI playstation* OR AB playstation* OR TI gamecube* OR AB gamecube* OR TI oculus OR AB oculus OR TI Kinect OR AB Kinect OR TI Kinects OR AB Kinects OR TI Gameboy* OR AB Gameboy* OR TI "game boy*" OR AB "game boy*" OR TI videogam* OR AB videogam* OR TI computergame* OR AB computergame* OR TI (technolog* N2 (game OR games OR gaming)) OR AB (technolog* N2 (game OR games OR gaming)) OR TI (program* N2 (game OR games OR gaming)) OR AB (program* N2 (game OR games OR gaming)) OR TI (electronic* N2 (game OR games OR gaming)) OR AB (electronic* N2 (game OR games OR gaming)) OR ((TI game OR AB game OR TI games OR AB games OR TI gaming* OR AB gaming* OR TI gamification* OR AB gamification* OR TI gamify* OR AB gamify* OR TI gamifie* OR AB gamifie* OR TI gamer* OR AB gamer*) AND (MH "virtual reality" OR MH "augmented reality" OR TI video OR AB video OR TI "mixed realit*" OR AB "mixed realit*" OR TI "AR" OR AB "AR" OR TI "VR" OR AB "VR" OR TI mobile OR AB mobile OR TI iphone* OR AB iphone* OR TI smartphone* OR AB smartphone* OR TI phone OR AB phone OR TI phones OR AB phones OR TI cellphone* OR AB cellphone* OR TI digital OR AB digital OR TI virtual OR AB virtual OR TI augmented OR AB augmented OR TI computer* OR AB computer* OR TI console AB console OR TI consoles OR AB consoles OR TI interactive AB interactive OR TI handheld* OR AB handheld* OR TI "hand held*" OR AB "hand held*" OR TI "PC" OR AB "PC" OR TI "PCs" OR AB "PCs" OR TI desktop OR AB desktop OR TI ipad OR AB ipad OR TI ipads OR AB ipads OR TI online OR AB online OR TI "on line" OR AB "on line" OR TI internet OR AB internet OR TI immers* OR AB immers* OR TI device* OR AB device* OR TI xbox OR AB xbox OR TI Xboxes OR AB Xboxes OR TI audiovisual OR AB audiovisual OR TI "audio visual" OR AB "audio visual" OR TI laptop* OR AB laptop* OR TI tablet OR AB tablet OR TI tablets OR AB tablets OR TI “smart glass*” OR AB “smart glass*” OR TI smartglass* OR AB smartglass* OR TI “google glass*” OR AB “google glass*” OR TI mhealth OR AB mhealth OR TI “m health” OR AB “m health”))) AND (MH (neoplasms+ OR "cancer fatigue" OR "cancer survivors" OR "cancer patients" OR "cancer pain" OR oncology+ OR "oncologic nursing+" OR "oncology care units" OR "rehabilitation, cancer" OR "oncologic care+" OR “early detection of cancer+” OR “cancer vaccines+”) OR TI (cancer* OR carcinoma* OR oncolog* OR psychooncolog* OR neoplas* OR tumor* OR tumour* OR malign*) OR AB (cancer* OR carcinoma* OR oncolog* OR psychooncolog* OR neoplas* OR tumor* OR tumour* OR malign*))

CINAHL Plus Full Text (EBSCOhost) Revised Search in Line by Line Format:

Note: Blue cells indicate results for a complete concept

Search No.	Search String	Results
S1	MH "video games+"	5,975
S2	TI "serious game" OR AB "serious game" OR TI "serious games" OR AB "serious games" OR TI "serious gaming" OR AB "serious gaming" OR TI "applied game" OR AB "applied game" OR TI "applied games" OR AB "applied games" OR TI "applied gaming" OR AB "applied gaming" OR TI exergam* OR AB exergam* OR TI "exer gaming" OR AB "exer gaming" OR TI "exer game*" OR AB "exer game*" OR TI Nintendo* OR AB Nintendo* OR TI Wii OR AB Wii OR TI Wiis OR AB Wiis OR TI playstation* OR AB playstation* OR TI gamecube* OR AB gamecube* OR TI oculus OR AB oculus OR TI Kinect OR AB Kinect OR TI Kinects OR AB Kinects OR TI Gameboy* OR AB Gameboy* OR TI "game boy*" OR AB "game boy*" OR TI videogam* OR AB videogam* OR TI computergame* OR AB computergame*	2,913
S3	S1 OR S2	7,508
S4	TI (technolog* N2 (game OR games OR gaming))	66
S5	AB (technolog* N2 (game OR games OR gaming))	175
S6	TI (program* N2 (game OR games OR gaming))	32
S7	AB (program* N2 (game OR games OR gaming))	171
S8	TI (electronic* N2 (game OR games OR gaming))	57
S9	AB (electronic* N2 (game OR games OR gaming))	209
S10	S3 OR S4 OR S5 OR S6 OR S7 OR S8 OR S9	7,875
S11	TI game OR AB game OR TI games OR AB games OR TI gaming* OR AB gaming* OR TI gamification* OR AB gamification* OR TI gamify* OR AB gamify* OR TI gamifie* OR AB gamifie* OR TI gamer* OR AB gamer*	23,556
S12	MH "virtual reality" OR MH "augmented reality"	8,628
S13	TI video OR AB video OR TI "mixed realit*" OR AB "mixed realit*" OR TI "AR" OR AB "AR" OR TI "VR" OR AB "VR" OR TI mobile OR AB mobile OR TI iphone* OR AB iphone* OR TI smartphone* OR AB smartphone* OR TI phone OR AB phone OR TI phones OR AB phones OR TI cellphone* OR AB cellphone* OR TI digital OR AB digital OR TI virtual OR AB virtual OR TI augmented OR AB augmented OR TI computer* OR AB computer* OR TI console AB console OR TI consoles OR AB consoles OR TI interactive AB interactive OR TI handheld* OR AB handheld* OR TI "hand held*" OR AB "hand held*" OR TI "PC" OR AB "PC" OR TI "PCs" OR AB "PCs" OR TI desktop OR AB desktop OR TI ipad OR AB ipad OR TI ipads OR AB ipads OR TI online OR AB online OR TI "on line" OR AB "on line" OR TI internet OR AB internet OR TI immers* OR AB immers* OR TI device* OR AB device* OR TI xbox OR AB xbox OR TI Xboxes OR AB Xboxes OR TI audiovisual OR AB audiovisual OR TI "audio visual" OR AB "audio visual" OR TI laptop* OR AB laptop* OR TI tablet OR AB tablet OR TI tablets OR AB tablets OR TI “smart glass*” OR AB “smart glass*” OR TI smartglass* OR AB smartglass* OR TI “google glass*” OR AB “google glass*” OR TI mhealth OR AB mhealth OR TI “m health” OR AB “m health”	587,419
S14	S12 OR S13	589,136
S15	S11 AND S14	8,770
**S16**	**S10 OR S15**	**12,693**
S17	MH (neoplasms+ OR "cancer fatigue" OR "cancer survivors" OR "cancer patients" OR "cancer pain" OR oncology+ OR "oncologic nursing+" OR "oncology care units" OR "rehabilitation, cancer" OR "oncologic care+" OR “early detection of cancer+” OR “cancer vaccines+”)	706,691

S18	TI (cancer* OR carcinoma* OR oncolog* OR psychooncolog* OR neoplas* OR tumor* OR tumour* OR malign*)	532,220
S19	AB (cancer* OR carcinoma* OR oncolog* OR psychooncolog* OR neoplas* OR tumor* OR tumour* OR malign*)	519,874
**S20**	**S17 OR S18 OR S19**	**941,288**
**S21**	**S16 AND S20**	**233**

Scopus: 1,168 results (12/15/2023)

Scopus Original Search in Block Format

(TITLE-ABS-KEY("serious game" OR "serious games" OR "serious gaming" OR "applied game" OR "applied games" OR "applied gaming" OR exergam* OR "exer gaming" OR "exer game*" OR Nintendo* OR Wii OR Wiis OR playstation* OR gamecube* OR oculus OR Kinect OR Kinects OR Gameboy* OR "game boy*" OR videogam*) OR TITLE-ABS-KEY(technolog* W/2 (game OR games OR gaming)) OR TITLE-ABS-KEY(program* W/2 (game OR games OR gaming)) OR TITLE-ABS-KEY(electronic* W/2 (game OR games OR gaming)) OR (TITLE-ABS-KEY(video OR "mixed realit*" OR "AR" OR "VR" OR mobile OR iphone* OR smartphone* OR phone OR phones OR cellphone* OR digital OR virtual OR augmented OR computer* OR console OR consoles OR interactive OR handheld* OR "hand held*" OR "PC" OR "PCs" OR desktop OR ipad OR ipads OR online OR "on line" OR internet OR immers* OR device* OR xbox OR Xboxes OR audiovisual OR "audio visual" OR laptop* OR tablet OR tablets) AND TITLE-ABS-KEY(game OR games OR gaming* OR gamification* OR gamify* OR gamifie* OR gamer*))) AND TITLE-ABS-KEY(cancer* OR carcinoma* OR oncolog* OR psychooncolog* OR neoplas* OR tumor* OR tumour* OR malign*)

Scopus Revised Search in Block Format

Note: This search was adapted from its original version (see the previous section, "Scopus Original Search", for the original version). 6 additional keywords were added. Curly brackets were also added around the "oculus" term to search it as an exact phrase, as Scopus was mapping the term to irrelevant terms like ocular (a mapping which, interestingly, hadn't occurred in the original search). All changes are indicated with yellow highlighting.

(TITLE-ABS-KEY("serious game" OR "serious games" OR "serious gaming" OR "applied game" OR "applied games" OR "applied gaming" OR exergam* OR "exer gaming" OR "exer game*" OR Nintendo* OR Wii OR Wiis OR playstation* OR gamecube* OR {oculus} OR Kinect OR Kinects OR Gameboy* OR "game boy*" OR videogam* OR computergame*) OR TITLE-ABS-KEY(technolog* W/2 (game OR games OR gaming)) OR TITLE-ABS-KEY(program* W/2 (game OR games OR gaming)) OR TITLE-ABS-KEY(electronic* W/2 (game OR games OR gaming)) OR (TITLE-ABS-KEY(video OR "mixed realit*" OR "AR" OR "VR" OR mobile OR iphone* OR smartphone* OR phone OR phones OR cellphone* OR digital OR virtual OR augmented OR computer* OR console OR consoles OR interactive OR handheld* OR "hand held*" OR "PC" OR "PCs" OR desktop OR ipad OR ipads OR online OR "on line" OR internet OR immers* OR device* OR xbox OR Xboxes OR audiovisual OR "audio visual" OR laptop* OR tablet OR tablets OR "smart glass*" OR smartglass* OR "google glass*" OR mhealth OR "m health") AND TITLE-ABS-KEY(game OR games OR gaming* OR gamification* OR gamify* OR gamifie* OR gamer*))) AND TITLE-ABS-KEY(cancer* OR carcinoma* OR oncolog* OR psychooncolog* OR neoplas* OR tumor* OR tumour* OR malign*)

Scopus Revised Search in Line by Line Format

Note: Blue cells indicate results for a complete concept

Search No.	Search String	Results
#1	TITLE-ABS-KEY("serious game" OR "serious games" OR "serious gaming" OR "applied game" OR "applied games" OR "applied gaming" OR exergam* OR "exer gaming" OR "exer game*" OR Nintendo* OR Wii OR Wiis OR playstation* OR gamecube* OR {oculus} OR Kinect OR Kinects OR Gameboy* OR "game boy*" OR videogam* OR computergame*)	39,086
#2	TITLE-ABS-KEY(technolog* W/2 (game OR games OR gaming))	4,411
#3	TITLE-ABS-KEY(program* W/2 (game OR games OR gaming))	3,527
#4	TITLE-ABS-KEY(electronic* W/2 (game OR games OR gaming))	1,502
#5	#1 OR #2 OR #3 OR #4	47,226
#6	TITLE-ABS-KEY(video OR "mixed realit*" OR "AR" OR "VR" OR mobile OR iphone* OR smartphone* OR phone OR phones OR cellphone* OR digital OR virtual OR augmented OR computer* OR console OR consoles OR interactive OR handheld* OR "hand held*" OR "PC" OR "PCs" OR desktop OR ipad OR ipads OR online OR "on line" OR internet OR immers* OR device* OR xbox OR Xboxes OR audiovisual OR "audio visual" OR laptop* OR tablet OR tablets OR “smart glass*” OR smartglass* OR “google glass*” OR mhealth OR “m health”)	13,163,617
#7	TITLE-ABS-KEY(game OR games OR gaming* OR gamification* OR gamify* OR gamifie* OR gamer*)	408,056
#8	#6 AND #7	173,605
**#9**	**#5 OR #8**	**197,689**
**#10**	**TITLE-ABS-KEY(cancer* OR carcinoma* OR oncolog* OR psychooncolog* OR neoplas* OR tumor* OR tumour* OR malign*)**	**6,388,563**
**#11**	**#9 AND #10**	**1,168**

Web of Science Core Collection: 757 results (12/15/2023)

Web of Science Core Collection Original Search in Block Format:

Instructions: In Web of Science, click "Advanced Search" below the search bar. Copy and paste into the "Query Preview" box. Click "Search."

(TS=("serious game" OR "serious games" OR "serious gaming" OR "applied game" OR "applied games" OR "applied gaming" OR exergam* OR "exer gaming" OR "exer game*" OR Nintendo* OR Wii OR Wiis OR playstation* OR gamecube* OR oculus OR Kinect OR Kinects OR Gameboy* OR "game boy*" OR videogam*) OR TS=(technolog* NEAR/2 (game OR games OR gaming)) OR TS=(program* NEAR/2 (game OR games OR gaming)) OR TS=(electronic* NEAR/2 (game OR games OR gaming)) OR (TS=(video OR "mixed realit*" OR "AR" OR "VR" OR mobile OR iphone* OR smartphone* OR phone OR phones OR cellphone* OR digital OR virtual OR augmented OR computer* OR console OR consoles OR interactive OR handheld* OR "hand held*" OR "PC" OR "PCs" OR desktop OR ipad OR ipads OR online OR "on line" OR internet OR immers* OR device* OR xbox OR Xboxes OR audiovisual OR "audio visual" OR laptop* OR tablet OR tablets) AND TS=(game OR games OR gaming* OR gamification* OR gamify* OR gamifie* OR gamer*))) AND TS=(cancer* OR carcinoma* OR oncolog* OR psychooncolog* OR neoplas* OR tumor* OR tumour* OR malign*)

Web of Science Core Collection Revised Search in Block Format

Note: This search was adapted from its original version (see the previous section, "Web of Science Core Collection Original Search", for the original version). 6 additional keywords were added. All changes are indicated with yellow highlighting.

(TS=("serious game" OR "serious games" OR "serious gaming" OR "applied game" OR "applied games" OR "applied gaming" OR exergam* OR "exer gaming" OR "exer game*" OR Nintendo* OR Wii OR Wiis OR playstation* OR gamecube* OR oculus OR Kinect OR Kinects OR Gameboy* OR "game boy*" OR videogam* OR computergame*) OR TS=(technolog* NEAR/2 (game OR games OR gaming)) OR TS=(program* NEAR/2 (game OR games OR gaming)) OR TS=(electronic* NEAR/2 (game OR games OR gaming)) OR (TS=(video OR "mixed realit*" OR "AR" OR "VR" OR mobile OR iphone* OR smartphone* OR phone OR phones OR cellphone* OR digital OR virtual OR augmented OR computer* OR console OR consoles OR interactive OR handheld* OR "hand held*" OR "PC" OR "PCs" OR desktop OR ipad OR ipads OR online OR "on line" OR internet OR immers* OR device* OR xbox OR Xboxes OR audiovisual OR "audio visual" OR laptop* OR tablet OR tablets OR "smart glass*" OR smartglass* OR "google glass*" OR mhealth OR "m health") AND TS=(game OR games OR gaming* OR gamification* OR gamify* OR gamifie* OR gamer*))) AND TS=(cancer* OR carcinoma* OR oncolog* OR psychooncolog* OR neoplas* OR tumor* OR tumour* OR malign*)

Web of Science Core Collection Revised Search in Line by Line Format:

Note: Blue cells indicate results for a complete concept

Search No.	Search String	Results
#1	TS=("serious game" OR "serious games" OR "serious gaming" OR "applied game" OR "applied games" OR "applied gaming" OR exergam* OR "exer gaming" OR "exer game*" OR Nintendo* OR Wii OR Wiis OR playstation* OR gamecube* OR oculus OR Kinect OR Kinects OR Gameboy* OR "game boy*" OR videogam* OR computergame*)	29,529
#2	TS=(technolog* NEAR/2 (game OR games OR gaming))	2,765
#3	TS=(program* NEAR/2 (game OR games OR gaming))	2,360
#4	TS=(electronic* NEAR/2 (game OR games OR gaming))	1,188
#5	#1 OR #2 OR #3 OR #4	34,929
#6	TS=(video OR "mixed realit*" OR "AR" OR "VR" OR mobile OR iphone* OR smartphone* OR phone OR phones OR cellphone* OR digital OR virtual OR augmented OR computer* OR console OR consoles OR interactive OR handheld* OR "hand held*" OR "PC" OR "PCs" OR desktop OR ipad OR ipads OR online OR "on line" OR internet OR immers* OR device* OR xbox OR Xboxes OR audiovisual OR "audio visual" OR laptop* OR tablet OR tablets OR “smart glass*” OR smartglass* OR “google glass*” OR mhealth OR “m health”)	6,713,242
#7	TS=(game OR games OR gaming* OR gamification* OR gamify* OR gamifie* OR gamer*)	332,609
#8	#6 AND #7	108,752
**#9**	**#5 OR #8**	**128,745**
**#10**	**TS=(cancer* OR carcinoma* OR oncolog* OR psychooncolog* OR neoplas* OR tumor* OR tumour* OR malign*)**	**5,126,262**
**#11**	**#9 AND #10**	**757**

PsycINFO (EBSCOhost): 111 results (12/15/2023)

PsycINFO (EBSCOhost) Original Search in Block Format:

(DE ("computer games" OR "digital gaming") OR TI "serious game" OR AB "serious game" OR TI "serious games" OR AB "serious games" OR TI "serious gaming" OR AB "serious gaming" OR TI "applied game" OR AB "applied game" OR TI "applied games" OR AB "applied games" OR TI "applied gaming" OR AB "applied gaming" OR TI exergam* OR AB exergam* OR TI "exer gaming" OR AB "exer gaming" OR TI "exer game*" OR AB "exer game*" OR TI Nintendo* OR AB Nintendo* OR TI Wii OR AB Wii OR TI Wiis OR AB Wiis OR TI playstation* OR AB playstation* OR TI gamecube* OR AB gamecube* OR TI oculus OR AB oculus OR TI Kinect OR AB Kinect OR TI Kinects OR AB Kinects OR TI Gameboy* OR AB Gameboy* OR TI "game boy*" OR AB "game boy*" OR TI videogam* OR AB videogam* OR TI (technolog* N2 game) OR TI (technolog* N2 games) OR TI (technolog* N2 gaming) OR TI (program* N2 game) OR TI (program* N2 games) OR TI (program* N2 gaming) OR TI (electronic* N2 game) OR TI (electronic* N2 games) OR TI (electronic* N2 gaming) OR AB (technolog* N2 game) OR AB (technolog* N2 games) OR AB (technolog* N2 gaming) OR AB (program* N2 game) OR AB (program* N2 games) OR AB (program* N2 gaming) OR AB (electronic* N2 game) OR AB (electronic* N2 games) OR AB (electronic* N2 gaming) OR ((TI game OR AB game OR TI games OR AB games OR TI gaming* OR AB gaming* OR TI gamification* OR AB gamification* OR TI gamify* OR AB gamify* OR TI gamifie* OR AB gamifie* OR TI gamer* OR AB gamer*) AND (DE "virtual reality" OR DE "augmented reality" OR TI video OR AB video OR TI "mixed realit*" OR AB "mixed realit*" OR TI "AR" OR AB "AR" OR TI "VR" OR AB "VR" OR TI mobile OR AB mobile OR TI iphone* OR AB iphone* OR TI smartphone* OR AB smartphone* OR TI phone OR AB phone OR TI phones OR AB phones OR TI cellphone* OR AB cellphone* OR TI digital OR AB digital OR TI virtual OR AB virtual OR TI augmented OR AB augmented OR TI computer* OR AB computer* OR TI console AB console OR TI consoles OR AB consoles OR TI interactive AB interactive OR TI handheld* OR AB handheld* OR TI "hand held*" OR AB "hand held*" OR TI "PC" OR AB "PC" OR TI "PCs" OR AB "PCs" OR TI desktop OR AB desktop OR TI ipad OR AB ipad OR TI ipads OR AB ipads OR TI online OR AB online OR TI "on line" OR AB "on line" OR TI internet OR AB internet OR TI immers* OR AB immers* OR TI device* OR AB device* OR TI xbox OR AB xbox OR TI Xboxes OR AB Xboxes OR TI audiovisual OR AB audiovisual OR TI "audio visual" OR AB "audio visual" OR TI laptop* OR AB laptop* OR TI tablet OR AB tablet OR TI tablets OR AB tablets))) AND (DE (neoplasms OR "endocrine neoplasms" OR leukemias OR melanoma OR metastasis OR "nervous system neoplasms" OR "brain neoplasms" OR glioma OR "terminal cancer" OR oncology) OR TI (cancer* OR carcinoma* OR oncolog* OR psychooncolog* OR neoplas* OR tumor* OR tumour* OR malign*) OR AB (cancer* OR carcinoma* OR oncolog* OR psychooncolog* OR neoplas* OR tumor* OR tumour* OR malign*))

PsycINFO (EBSCOhost) Revised Search in Block Format

Note: This search was adapted from its original version (see the previous section, "PsycINFO Original Search", for the original version). 2 index terms and 6 additional keywords were added. All changes are indicated with yellow highlighting.

(DE ("computer games" OR "digital gaming" OR "digital game-based learning") OR TI "serious game" OR AB "serious game" OR TI "serious games" OR AB "serious games" OR TI "serious gaming" OR AB "serious gaming" OR TI "applied game" OR AB "applied game" OR TI "applied games" OR AB "applied games" OR TI "applied gaming" OR AB "applied gaming" OR TI exergam* OR AB exergam* OR TI "exer gaming" OR AB "exer gaming" OR TI "exer game*" OR AB "exer game*" OR TI Nintendo* OR AB Nintendo* OR TI Wii OR AB Wii OR TI Wiis OR AB Wiis OR TI playstation* OR AB playstation* OR TI gamecube* OR AB gamecube* OR TI oculus OR AB oculus OR TI Kinect OR AB Kinect OR TI Kinects OR AB Kinects OR TI Gameboy* OR AB Gameboy* OR TI "game boy*" OR AB "game boy*" OR TI videogam* OR AB videogam* OR TI computergame* OR AB computergame* OR TI (technolog* N2 game) OR TI (technolog* N2 games) OR TI (technolog* N2 gaming) OR TI (program* N2 game) OR TI (program* N2 games) OR TI (program* N2 gaming) OR TI (electronic* N2 game) OR TI (electronic* N2 games) OR TI (electronic* N2 gaming) OR AB (technolog* N2 game) OR AB (technolog* N2 games) OR AB (technolog* N2 gaming) OR AB (program* N2 game) OR AB (program* N2 games) OR AB (program* N2 gaming) OR AB (electronic* N2 game) OR AB (electronic* N2 games) OR AB (electronic* N2 gaming) OR ((TI game OR AB game OR TI games OR AB games OR TI gaming* OR AB gaming* OR TI gamification* OR AB gamification* OR TI gamify* OR AB gamify* OR TI gamifie* OR AB gamifie* OR TI gamer* OR AB gamer*) AND (DE "virtual reality" OR DE "augmented reality" OR TI video OR AB video OR TI "mixed realit*" OR AB "mixed realit*" OR TI "AR" OR AB "AR" OR TI "VR" OR AB "VR" OR TI mobile OR AB mobile OR TI iphone* OR AB iphone* OR TI smartphone* OR AB smartphone* OR TI phone OR AB phone OR TI phones OR AB phones OR TI cellphone* OR AB cellphone* OR TI digital OR AB digital OR TI virtual OR AB virtual OR TI augmented OR AB augmented OR TI computer* OR AB computer* OR TI console AB console OR TI consoles OR AB consoles OR TI interactive AB interactive OR TI handheld* OR AB handheld* OR TI "hand held*" OR AB "hand held*" OR TI "PC" OR AB "PC" OR TI "PCs" OR AB "PCs" OR TI desktop OR AB desktop OR TI ipad OR AB ipad OR TI ipads OR AB ipads OR TI online OR AB online OR TI "on line" OR AB "on line" OR TI internet OR AB internet OR TI immers* OR AB immers* OR TI device* OR AB device* OR TI xbox OR AB xbox OR TI Xboxes OR AB Xboxes OR TI audiovisual OR AB audiovisual OR TI "audio visual" OR AB "audio visual" OR TI laptop* OR AB laptop* OR TI tablet OR AB tablet OR TI tablets OR AB tablets OR TI "smart glass*" OR AB "smart glass*" OR TI smartglass* OR AB smartglass* OR TI "google glass*" OR AB "google glass*" OR TI mhealth OR AB mhealth OR TI "m health" OR AB "m health"))) AND (DE (neoplasms OR "endocrine neoplasms" OR leukemias OR melanoma OR metastasis OR "nervous system neoplasms" OR "brain neoplasms" OR glioma OR "terminal cancer" OR oncology OR "cancer screening") OR TI (cancer* OR carcinoma* OR oncolog* OR psychooncolog* OR neoplas* OR tumor* OR tumour* OR malign*) OR AB (cancer* OR carcinoma* OR oncolog* OR psychooncolog* OR neoplas* OR tumor* OR tumour* OR malign*))

PsycINFO (EBSCOhost) Revised Search in Line by Line Format:

Note: Blue cells indicate results for a complete concept

Search No.	Search String	Results
S1	DE ("computer games" OR "digital gaming" OR "digital game-based learning")	11,740
S2	TI "serious game" OR AB "serious game" OR TI "serious games" OR AB "serious games" OR TI "serious gaming" OR AB "serious gaming" OR TI "applied game" OR AB "applied game" OR TI "applied games" OR AB "applied games" OR TI "applied gaming" OR AB "applied gaming" OR TI exergam* OR AB exergam* OR TI "exer gaming" OR AB "exer gaming" OR TI "exer game*" OR AB "exer game*" OR TI Nintendo* OR AB Nintendo* OR TI Wii OR AB Wii OR TI Wiis OR AB Wiis OR TI playstation* OR AB playstation* OR TI gamecube* OR AB gamecube* OR TI oculus OR AB oculus OR TI Kinect OR AB Kinect OR TI Kinects OR AB Kinects OR TI Gameboy* OR AB Gameboy* OR TI "game boy*" OR AB "game boy*" OR TI videogam* OR AB videogam* OR TI computergame* OR AB computergame*	3,545
S3	TI (technolog* N2 game)	85
S4	TI (technolog* N2 games)	85
S5	TI (technolog* N2 gaming)	20
S6	TI (program* N2 game)	71
S7	TI (program* N2 games)	71
S8	TI (program* N2 gaming)	7
S9	TI (electronic* N2 game)	68
S10	TI (electronic* N2 games)	68
S11	TI (electronic* N2 gaming)	83
S12	AB (technolog* N2 game)	343
S13	AB (technolog* N2 games)	343
S14	AB (technolog* N2 gaming)	126
S15	AB (program* N2 game)	412
S16	AB (program* N2 games)	412
S17	AB (program* N2 gaming)	32
S18	AB (electronic* N2 game)	264
S19	AB (electronic* N2 games)	264
S20	AB (electronic* N2 gaming)	231
S21	S1 OR S2 OR S3 OR S4 OR S5 OR S6 OR S7 OR S8 OR S9 OR S10 OR S11 OR S12 OR S13 OR S14 OR S15 OR S16 OR S17 OR S18 OR S19 OR S20 OR S21	14,334
S22	TI game OR AB game OR TI games OR AB games OR TI gaming* OR AB gaming* OR TI gamification* OR AB gamification* OR TI gamify* OR AB gamify* OR TI gamifie* OR AB gamifie* OR TI gamer* OR AB gamer*	55,441
S23	DE ("virtual reality" OR "augmented reality")	12,514
S24	TI video OR AB video OR TI "mixed realit*" OR AB "mixed realit*" OR TI "AR" OR AB "AR" OR TI "VR" OR AB "VR" OR TI mobile OR AB mobile OR TI iphone* OR AB iphone* OR TI smartphone* OR AB smartphone* OR TI phone OR AB phone OR TI phones OR AB phones OR TI cellphone* OR AB cellphone* OR TI digital OR AB digital OR TI virtual OR AB virtual OR TI augmented OR AB augmented OR TI computer* OR AB computer* OR TI console AB console OR TI consoles OR AB consoles OR TI interactive AB interactive OR TI handheld* OR AB handheld* OR TI "hand held*" OR AB "hand held*" OR TI "PC" OR AB "PC" OR TI "PCs" OR AB "PCs" OR TI desktop OR AB desktop OR TI ipad OR AB ipad OR TI ipads OR AB ipads OR TI online OR AB online OR TI "on line" OR AB "on line" OR TI internet OR AB internet OR TI immers* OR AB immers* OR TI device* OR AB device* OR TI xbox OR AB xbox OR TI Xboxes OR AB Xboxes OR TI audiovisual OR AB audiovisual OR TI "audio visual" OR AB "audio visual" OR TI laptop* OR AB laptop* OR TI tablet OR AB tablet OR TI tablets OR AB tablets OR TI	503,649

	“smart glass*” OR AB “smart glass*” OR TI smartglass* OR AB smartglass* OR TI “google glass*” OR AB “google glass*” OR TI mhealth OR AB mhealth OR TI “m health” OR AB “m health”	
S25	S23 OR S24	504,029
S26	S22 AND S25	22,312
**S27**	**S21 OR S26**	**26,217**
S28	DE (neoplasms OR "endocrine neoplasms" OR leukemias OR melanoma OR metastasis OR "nervous system neoplasms" OR "brain neoplasms" OR glioma OR "terminal cancer" OR oncology OR “cancer screening”)	60,517
S29	TI (cancer* OR carcinoma* OR oncolog* OR psychooncolog* OR neoplas* OR tumor* OR tumour* OR malign*)	53,226
S30	AB (cancer* OR carcinoma* OR oncolog* OR psychooncolog* OR neoplas* OR tumor* OR tumour* OR malign*)	94,284
**S31**	**S28 OR S29 OR S30**	**101,723**
**S32**	**S27 AND S31**	**111**
